# *Saccharomyces cerevisiae* as a Model to Study Replicative Senescence Triggered by Telomere Shortening

**DOI:** 10.3389/fonc.2013.00101

**Published:** 2013-04-26

**Authors:** M. Teresa Teixeira

**Affiliations:** ^1^Laboratoire de Biologie Moléculaire et Cellulaire des Eucaryotes, FRE3354 Centre National de la Recherche Scientifique, Université Pierre et Marie Curie, Institut de Biologie Physico-ChimiqueParis, France

**Keywords:** telomeres, telomerase, DNA replication, DNA damage checkpoints, *Saccharomyces cerevisiae*, replicative senescence

## Abstract

In many somatic human tissues, telomeres shorten progressively because of the DNA-end replication problem. Consequently, cells cease to proliferate and are maintained in a metabolically viable state called replicative senescence. These cells are characterized by an activation of DNA damage checkpoints stemming from eroded telomeres, which are bypassed in many cancer cells. Hence, replicative senescence has been considered one of the most potent tumor suppressor pathways. However, the mechanism through which short telomeres trigger this cellular response is far from being understood. When telomerase is removed experimentally in *Saccharomyces cerevisiae*, telomere shortening also results in a gradual arrest of population growth, suggesting that replicative senescence also occurs in this unicellular eukaryote. In this review, we present the key steps that have contributed to the understanding of the mechanisms underlying the establishment of replicative senescence in budding yeast. As in mammals, signals stemming from short telomeres activate the DNA damage checkpoints, suggesting that the early cellular response to the shortest telomere(s) is conserved in evolution. Yet closer analysis reveals a complex picture in which the apparent single checkpoint response may result from a variety of telomeric alterations expressed in the absence of telomerase. Accordingly, the DNA replication of eroding telomeres appears as a critical challenge for senescing budding yeast cells and the easy manipulation of *S. cerevisiae* is providing insights into the way short telomeres are integrated into their chromatin and nuclear environments. Finally, the loss of telomerase in budding yeast triggers a more general metabolic alteration that remains largely unexplored. Thus, telomerase-deficient *S. cerevisiae* cells may have more common points than anticipated with somatic cells, in which telomerase depletion is naturally programed, thus potentially inspiring investigations in mammalian cells.

## Introduction

**Figure 1:**
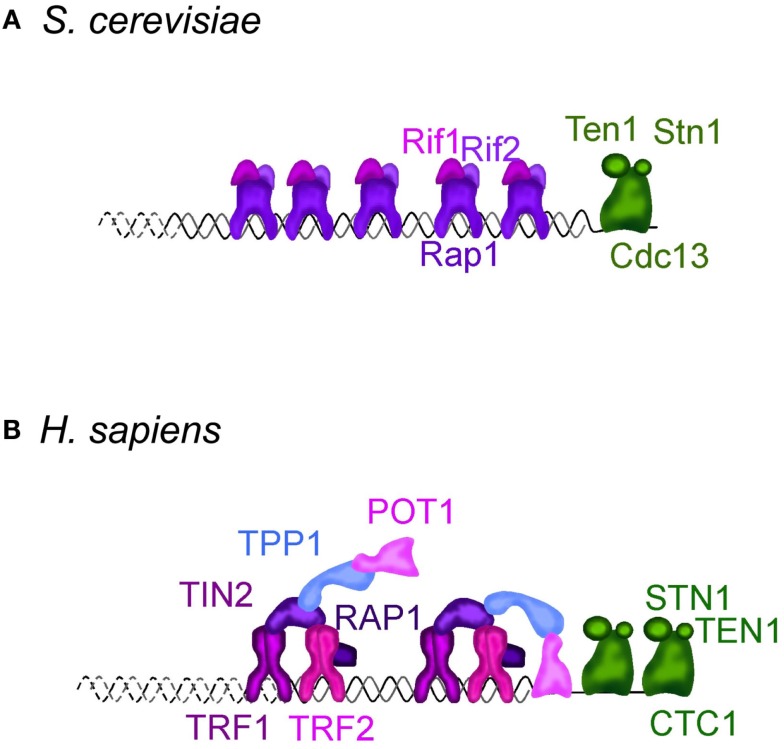
**Telomere structure in *S. cerevisiae* (A) and *H. sapiens* (B)**. **(A)** *S. cerevisiae* telomeric dsDNA is coated by Rap1, that tethers Rif1 and Rif2, two important regulators of telomere length homeostasis and telomere protectors. Sir2-4 also bind Rap1, but are not represented. The ssDNA is bound by Cdc13, which interacts with Stn1 and Ten1 forming a complex called CST. It protects the telomeres, regulates the overhang length and telomere length homeostasis. More details can be found in Wellinger and Zakian (2012). **(B)** In *H. sapiens* and other mammals, telomeric DNA is coated by a complex dubbed “shelterin” composed of TRF1, TRF2, RAP1, TIN2, TPP1, and POT1. TRF1 and TRF2 share with budding yeast Rap1 the DNA binding domain. Mammalian RAP1 ortholog is not directly bound to telomeric DNA, but is associated through an interaction with TRF2. TIN2, TPP1, and POT1 have no obvious orthologs in *S. cerevisiae*. CTC1, STN1, and TEN1 compose the CST complex in *H. sapiens*. Although distantly related, CST also protects the telomeres, regulates telomerase, and is involved in DNA replication, including at non-telomeric sites. More details in Palm and de Lange (2008), Giraud-Panis et al. (2010), Jain and Cooper (2010).

**Figure 2:**
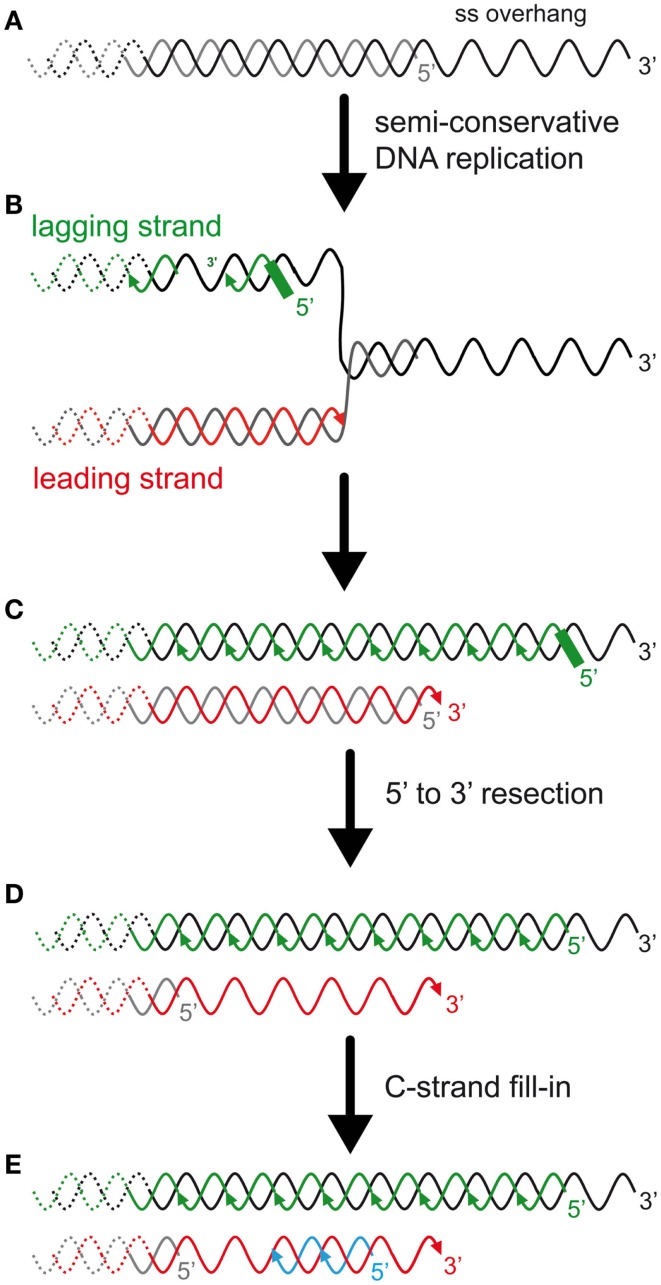
**The DNA-end replication problem in eukaryotes and the rate of telomere shortening**. **(A)** In many eukaryotes, including budding yeast and humans, telomeres end with a 3′ single-stranded overhang. **(B)** Most telomeric DNA is replicated by replication forks that arise unidirectionally from subtelomeric elements (Miller et al., 2006; Makovets, 2009; Sfeir et al., 2009). Thus, most TG-rich strands, containing the 3′ end, are replicated by the lagging synthesis machinery (green), and most CA-rich strands, containing the 5′ end, are replicated by the leading synthesis machinery (red). **(C)** The presence of the overhang implies an asymmetry between the two template strands and is expected to result in an asymmetry in the length of the semi-conservative DNA replication products. On one hand, the lagging strand telomere is expected to conserve the longest strand containing the 3′ end (but this has not been addressed experimentally). The 5′ newly synthesized lagging strand starts with an RNA primer of a few ribonucleotides. The positioning of this last Okazaki fragment and subsequent removal of the RNA primer is expected to determine the length of the overhang of this strand. In mammals, the positioning of the first RNA primer is random and its removal is delayed (Chow et al., 2012). The enzyme(s) involved in the RNA primer maturation are currently unknown. On another hand, the leading strand synthesis (red) presumably stops prematurely due to a lack of template that corresponds to the length of the overhang. Thus, this telomere is shorter than the parental telomere and the lagging telomere. **(D)** 5′–3′ Resection of the CA-rich strands was shown to occur in both leading and lagging telomeres in mammals (Wu et al., 2012). In *S. cerevisiae*, this resection is probably limited to the leading telomere, as represented (Faure et al., 2010). **(E)** C-strand fill-in compensates for this resection. This synthesis likely requires the polymerase alpha/primase with the synthesis of an RNA primer that must be processed, similarly to the lagging telomere. Thus, the generation of the overhangs is different between leading and lagging telomeres and these processes are expected to modulate telomere-shortening rate and subsequently the onset of senescence.

Telomeres are the ends of linear chromosomes, and in many eukaryotes they consist of a variable number of repetitive small sequences of a GT-rich motif running from the 5′ to the 3′ protruding end. Telomeres are coated with a specific set of proteins, both in their double-stranded region and in their single-stranded DNA extremity (Figure 1). In mammals, a part of these has been dubbed “shelterin” for its role in protecting chromosome ends from fusions and degradations (Palm and de Lange, 2008). Because of the inability of the semi-conservative DNA replication machinery to fully replicate DNA extremities, telomeric sequences are lost at each passage of the replication fork (Watson, 1972; Olovnikiv, 1973; Lingner et al., 1995) (Figure 2). For this reason, telomeres have been considered molecular clocks that count the number of divisions of individual cells. Telomerase is a reverse transcriptase that compensates for these sequence losses by synthesizing telomeric repeats using an associated non-coding RNA, the telomerase RNA, as a template. Most eukaryote species express telomerase for their long-term maintenance, but in some tissues of multicellular organisms, for instance most somatic cells of humans, telomerase can be downregulated and telomeres shorten as cells divide and differentiate. This telomere shortening leads to an irreversible cell division arrest called replicative senescence, or to apoptosis. Although replicative senescence can be achieved by other means depending on the species and tissues, the cellular response to short telomeres is now considered one of the most potent barriers to cancer emergence. Its role in aging is more controversial, although the mean telomere length decreases with age, and senescent cells accumulate in older human individuals and other mammals (Lansdorp, 2008). For all of these reasons it is important to understand how telomeres shorten and how short telomeres trigger a crucial signal for the fate of cells and the organism. Moreover, all eukaryotes possess linear chromosomes. Whether this is an advantage to control cell proliferation, a prerequisite to multicellularity or simply a way to cope with large genomes, is an evolutionary question that one can only speculate on.

Several model organisms and systems have been used to address these questions. The focus of this review is to describe how *Saccharomyces cerevisiae* has served to improve our understanding of how telomeres regulate and control cell proliferation potential. The key experiments demonstrating that a DNA damage checkpoint emanating from eroded telomeres is activated in telomerase-negative budding yeast cells are presented. This is followed by a discussion on the possible structures at telomeres that may cause this signal and how cells respond at molecular level.

## Telomere-Induced Replicative Senescence in Mammals: The Facts and the Questions

Replicative senescence was first described as the proliferative limit of human diploid fibroblasts when cultured *ex vivo* (Hayflick, 1965). Structural analysis of cells having reached this so-called Hayflick limit led to the definition of senescence as cells that cannot divide despite being supplied with sufficient nutrients and physiological stimuli such as growth factors or mitogens. Cells present a change in their shape and transcriptional profile and their secretion program is altered (Campisi and d’Adda di Fagagna, 2007). Their DNA is more condensed with the appearance of senescence-associated heterochromatin foci (SAHFs). Mammalian cells that reach this state in culture usually show a G1 DNA content, although primary arrest for some might occur in G2/M (Pignolo et al., 1998; Campisi and d’Adda di Fagagna, 2007; Jullien et al., 2012; Mao et al., 2012).

Although several stimuli can lead to senescence, telomere attrition with passage or age is the most extensively studied. This telomere attrition is due to the so-called DNA-end replication problem that is caused by the inability of DNA extremities to be fully duplicated (see Figure 2). This leads to a progressive telomere shortening at each passage of the replication fork, which can be reversed by re-expression of telomerase (Harley et al., 1990; Bodnar et al., 1998). This telomere erosion presumably causes a progressive alteration of the properties of the protective structures of natural chromosome ends. Accordingly, telomeres in these cells are recognized by several DNA damage recognition factors and repairing activities to form foci termed telomere-induced foci (TIFs). The implicated pathway is a cascade of phosphorylations, effected by the DNA damage checkpoints, starting with the phospho-inositide-3 kinase-related protein kinase (PIKK) ataxia telangiectasia mutated (ATM) and ataxia telangiectasia- and Rad3-related protein kinase (ATR), and their downstream substrates, the kinases CHK1 and CHK2. This leads to the phosphorylation of the transcription factor p53 and expression of the kinase p21, whose targets, among other things, inhibit cell cycle progression (Deng et al., 2008). Therefore, the current model in primary fibroblasts is that short telomeres, because they have lost most of the shelterin components that protect them, resemble accidental DNA double-strand breaks (DSBs) (Karlseder et al., 2002; d’Adda di Fagagna et al., 2003; Deng et al., 2008). Yet the full DNA repair is not triggered at damaged telomeres (Fumagalli et al., 2012), suggesting that short telomeres may display properties that remain to be understood.

Telomere shortening and induction of a DNA damage checkpoint at telomeres is also likely occurring physiologically in proliferative human tissues *in vivo* in which telomerase activity is down regulated (Hastie et al., 1990; Jeyapalan and Sedivy, 2008). Indeed, several studies show that telomeres shorten with the age of individuals and TIFs form in dermal fibroblasts of old primates (Herbig et al., 2006; Jeyapalan et al., 2007). Interestingly, in most of these cells, a single TIF is observed. Although a single TIF could contain several short telomeres, this raises the possibility that a single short telomere reaching a critical length is sufficient to generate a signal able to recruit a sufficient amount of DNA-repair factors to be visualized. This contrasts to the situation in cultured cells where several short telomeres could be required to trigger senescence (Jeyapalan et al., 2007; Kaul et al., 2011).

While the relevance of the findings using cell culture to what happens in whole organisms during aging is growing, several notions remain elusive. For instance, are short telomeres, which have retained a few telomeric repeats, recognized as DSBs or is there a distinct telomeric signal(s)? Is a short telomere a dysfunctional telomere, or does it accomplish a specific function to stop cell division and prevent genomic instability at the same time? What type of DNA repair is triggered by eroded telomeres? What are the risks of genomic instability for the cell? As we are going to see in the next sections, the early response of budding yeast to progressive telomere shortening is strikingly similar to the one described so far in fibroblasts and other mammalian systems. Likewise, in this model organism, the response is more complex than a simple response to DSBs. Furthermore, other prominent questions concerning the cellular consequences of replicative senescence in humans may find unexpected answers in non-biased experimental approaches in yeast.

## *S. cerevisiae* as a Model Organism for Telomere Biology

Perhaps because of the history of their discovery and their first molecular description in ciliates, and perhaps because of their involvement in several steps of infection by protozoa, telomeres have been studied at length in a remarkable range of eukaryotes, covering most eukaryote supergroups (Figueiredo and Scherf, 2005; Lira et al., 2007; Zakian, 2012). One striking observation is that telomeres share the same common function of chromosome end protection and maintenance through a more or less conserved architecture of protein complexes (Figure 1) (Teixeira and Gilson, 2005; Linger and Price, 2009; Giraud-Panis et al., 2010). Therefore, it is tempting to speculate that the cellular response to short telomeres is also conserved.

Yeasts are a polyphyletic group of unicellular organisms that have emerged repeatedly from filamentous fungi (Keeling et al., 2005; Dujon, 2010). With animals, they share a common evolutionary origin that gave rise to the Opisthokonts phylum, representing a small portion of all eukaryotic diversity. Because of its easy manipulation and powerful genetic engineering, *S. cerevisiae* entered the scene as a tool for the study of telomeres very early in the history of telomere molecular biology (Szostak and Blackburn, 1982). Very rapidly, it also became a model organism for the understanding of telomere maintenance and for the identification of telomerase (Shampay et al., 1984; Lingner et al., 1997b). Finally, the milestone work that paved the way for the genetic study of telomeres and telomerase in budding yeast was an ingenious genetic screen that led to the isolation of mutations in the telomere maintenance pathway that were revealed later to be mutations of telomerase activity (Lundblad and Szostak, 1989; Lendvay et al., 1996; Lingner et al., 1997a,b). These mutants, called *EST1-4* (ever short telomere), exhibited progressive telomere shortening and, strikingly, lost viability after about 60–80 generations. Late cultures of these strains eventually regained proliferation potential. A small subset of cells, so-called post-senescence survivors, could emerge that maintain telomeres through telomerase-independent means, notably by homologous recombination (HR) (Lundblad and Blackburn, 1993). *TLC1*, the gene encoding the RNA template component of telomerase, also found independently in a genetic screen designed to investigate silencing at telomeres, displayed the same phenotype when mutated (Singer and Gottschling, 1994). These experiments showed that, similar to human fibroblasts, yeast cells could survive in the short term without telomerase. As we will see, this time is long enough to study the telomere-shortening mechanism and the cellular consequences of telomerase loss. Later on, *EST2* was shown to encode the catalytic subunit of telomerase and *EST1* and *EST3*, two components of telomerase holoenzyme essential for *in vivo* activity (Lingner et al., 1997a,b; Hughes et al., 2000).

In a first attempt to study telomere function in *S. cerevisiae*, a first critical experiment was to remove a telomere from its chromosome end (Sandell and Zakian, 1993). In order to precisely dissect the cellular response to a lack of a single telomere and to distinguish it from a lack of essential genes in case of potential chromosome instability, a unique recognition site for the endonuclease HO was introduced 20 kb away from the end of a non-essential chromosome. This was achieved by the generation of a haploid strain containing this chromosome as a disome. After the conditional induction of HO, cells were found to immediately arrest in a *RAD9*-dependent manner. This was strikingly similar to a DSB, which had been demonstrated by that time to provoke a G2/M arrest, defined as a checkpoint (Weinert and Hartwell, 1988). The authors concluded that telomeres are essential in preventing DNA damage checkpoints from being activated at natural chromosome ends.

Tools to physically characterize *S. cerevisiae* telomeres, namely to determine global length and 3′-overhang quantity, were subsequently rapidly developed, contributing to a gradual increase in knowledge on telomere structure and maintenance. The 16 *S. cerevisiae* chromosomes end with 275–375 bp of a degenerated motif TG_1–3_/C_1–3_A (see Wellinger and Zakian, 2012 for an exhaustive recent review). These are followed internally by a long gene-poor region containing subtelomeric repeated elements X and Y′ of several thousands of base pairs. Rap1 is the major telomere-binding protein. It shares similarities with some of the mammalian shelterin components and binds double-stranded telomeric DNA through the conserved Myb domain (Teixeira and Gilson, 2005; Lue, 2010). Rap1 tethers several additional factors involved in essential telomeric functions, such as Rif1 and Rif2, important regulators of telomerase activity and telomere protectors. It also interacts with Sir2, Sir3, and Sir4, which are more specialized in telomere chromatin establishment. The single-stranded G-rich tail was estimated to 12–15 nt long and increases to up 300 nt at the end of S-phase, by the time telomeres are replicated in *S. cerevisiae* (Wellinger et al., 1993; Dionne and Wellinger, 1996, 1998) (Figure 2). The telomeric ssDNA is bound by Cdc13, which, like other telomere single-strand binding proteins in other species, possesses an oligosaccharide binding-fold motif. Cdc13 can be found in complex with Stn1 and Ten1 to form a trimer called CST. What is interesting is that the tridimensional structure of CST resembles the major ssDNA binding ternary complex replication protein A (RPA), which is involved in the binding of the lagging strand template during semi-conservative DNA replication (Gelinas et al., 2009). Cdc13 has a dual activity at telomeres: it interacts with telomerase holoenzyme to promote telomere elongation, and, in complex with Ten1 and Stn1, it promotes C-strand synthesis through an interaction with the DNA polymerase alpha/primase (Nugent et al., 1996; Diede and Gottschling, 1999; Qi and Zakian, 2000; Grandin et al., 2001; Giraud-Panis et al., 2010). Moreover, possibly related to the latter function, the CST complex is involved in protecting the DNA ends from exonucleases and recombination. Overall, the telomere architecture in *S. cerevisiae* conserves most of the functional protein modules observed in other eukaryotes.

## Brief Overview of the DNA Damage Response in *S. cerevisiae*

**Figure 3:**
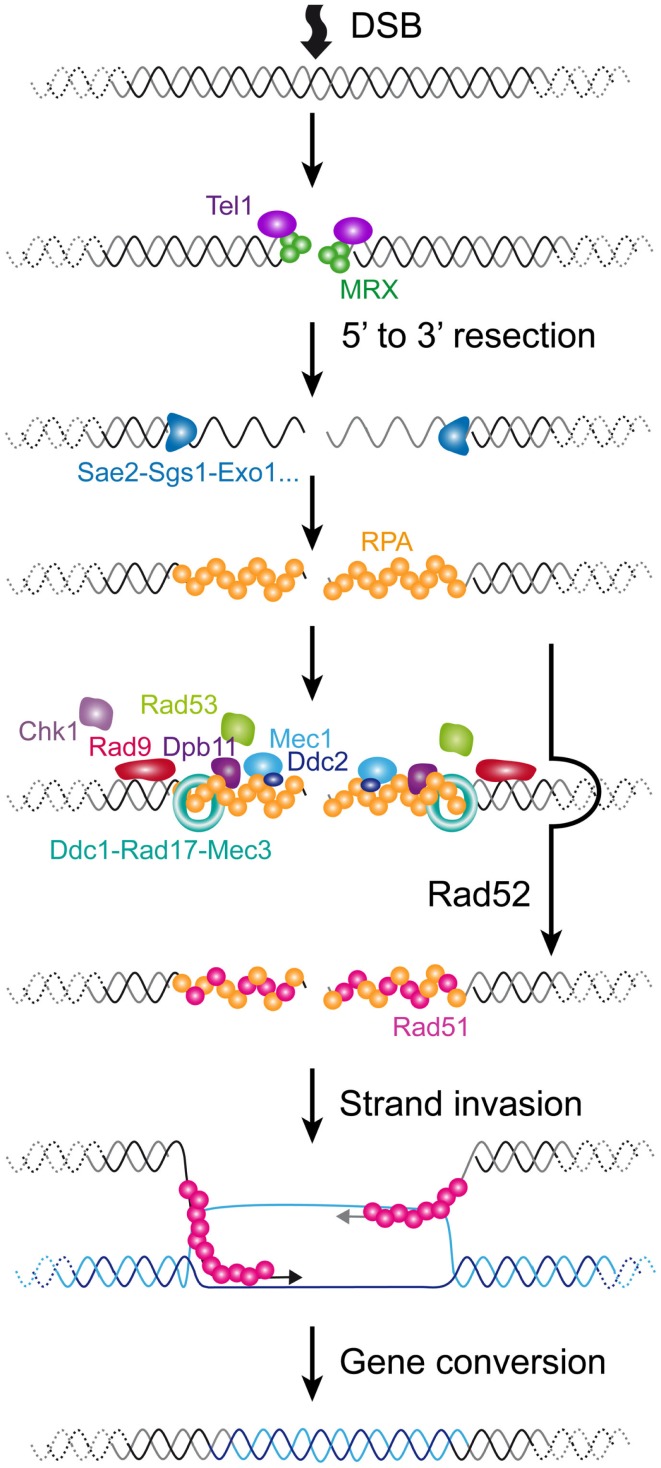
**Cellular responses to a DSB in *S. cerevisiae***. A DSB is rapidly recognized by MRX that recruits Tel1. In G1 phase of the cell cycle, the DSB is repaired mainly by NHEJ (not represented). In S/G2 phases of the cell cycle, the DSB is processed for repair by HR. The 5′–3′ resection is achieved by the concerted action of nucleases and helicases including Sae2, Sgs1, Dna2, and Exo1. The resulting ssDNA is then coated by RPA. In yeast, Rad52 then catalyzes the displacement of RPA by Rad51. The resulting Rad51 filament can then initiate homology search and strand invasion and prime DNA synthesis. Resolution of DNA structure is then required to conclude the gene conversion. The DNA checkpoint pathway starts by Tel1 recruitment, which modifies nearby nucleosomes (not represented) to form a first platform to recruit additional checkpoint components. The RPA-coated ssDNA forms a second platform that recruits Ddc2-Mec1, Dpb11, and the 9-1-1 checkpoint clamp (Ddc1-Rad17-Mec3), assisted by the checkpoint clamp loader (Rad24-Rfc2-5, not represented). Rad9 is then recruited and activated. Collectively, this edifice contributes to the efficient activation of Rad53 and Chk1 that will phosphorylate downstream targets.

The current model of senescence is that, as they shorten, telomeres resemble a DSB. Thus, before getting into the detailed comparison of short telomeres and DNA damage, it is worth recalling briefly the current knowledge on the DNA damage response, particularly on DSBs. This pathway is remarkably conserved in evolution, and we will only provide a small glimpse of the vast literature in this area focusing on *S. cerevisiae*, which we scrutinize in this review (see Figure 3; Table 1). More detailed information on this subject can be found elsewhere (Harrison and Haber, 2006; Heyer et al., 2010; Finn et al., 2012).

**Table 1:** **Homologs of the major components of the DNA damage repair pathway**.

*S. cerevisiae*	*S. pombe*	*H. sapiens*	Brief description
Mre11-Rad50-Xrs2	Rad32-Rad50-Nbs1	MRE11-RAD50-NBS1	DSB sensor
Yku70-Yku80	Pku70-Pku80	KU70-KU80	DSB sensor
Tel1	Tel1	ATM	Protein kinase (PIKK)
Sae2	Ctp1	CtIP	Endonuclease
Exo1	Exo1	EXO1	5′–3′ Exonuclease and flap-endonuclease
Sgs1	Rqh1	BLM	DNA helicase
Dna2	Dna2	DNA2	DNA-dependent ATPase, ATP-dependent nuclease, and helicase
Mec1-Ddc2	Rad3-Rad26	ATR-ATRIP	Protein kinase (PIKK) and interacting factor
Rad24-Rfc2-5	Rad17-Rfc2-5	RAD17-RFC2-5	Clamp loader; RFC-like complex
Ddc1-Rad17-Mec3	Rad9-Rad1-Hus1	RAD9-RAD1-HUS1	Checkpoint clamp; PCNA-like; also called 9-1-1 complex
Dpb11	Cut5	TopBP1	Replication initiation protein and checkpoint sensor
Rad9	Crb2	53BP1; BRCA1; MDC1	Checkpoint adaptors/mediators
Mrc1	Mrc1	Claspin	Checkpoint mediator and replication fork component
Rad53	Cds1	CHK2	Protein kinase; checkpoint effector
Chk1	Chk1	CHK1	Protein kinase; checkpoint effector

Double-strand breaks can occur at any time of the cell cycle because of direct exogenous injuries and or as a result of replication fork collapse. The first step in a DSB is the recruitment of the complex composed of Mre11-Rad50-Xrs2 (MRX) and the Ku complex composed of Yku70-Yku80. MRX then recruits Tel1 (ATM in mammals). In the G1 phase of the cell cycle, these DSBs are repaired via the non-homologous end-joining (NHEJ) pathway, which leads to the ligation of the broken ends by specific ligase activities. In the S/G2 phases of the cell cycle, high CDK activity and downregulation of NHEJ factors lead to the phosphorylation of certain targets, which favors the rapid resection of the 5′ end strands (Ira et al., 2004; Huertas et al., 2008; Zierhut and Diffley, 2008). This results in the formation of long single-stranded 3′ overhangs. Resection involves the MRX complex itself jointly with Sae2 for a first step and Sgs1, Dna2, and Exo1 for extended resected products (Mimitou and Symington, 2008). Subsequently, RPA, the main ssDNA binding protein in eukaryotes, accumulates, forming a platform for the recruitment of many repair factors. A major repair pathway in *S. cerevisiae* is HR, which requires the recruitment of Rad51, enabling the process of homology search. Rad52 assists the loading of Rad51 in *S. cerevisiae* and plays a central role in HR in this species, being involved in several HR pathways and being responsible for the most severe phenotype when mutated (San Filippo et al., 2008).

In parallel with these events, cells activate the DNA damage checkpoints (reviewed in Finn et al., 2012). Checkpoints were first defined as factors that contribute to an arrest in cell cycle progression in the presence of damage (Weinert and Hartwell, 1988). They are important for cell viability because they give cells time to repair the damage by preventing them from entering the next cell cycle phase with deleterious damage. Factors involved are characterized by a high degree of genotoxic cell sensitivity when they are mutated. However, in contrast to mutations in a repair factor, mutations in checkpoints do not delay or arrest the cell cycle immediately after the DNA damage. Instead of arresting, cells undergo a few cell divisions before dying (Hartwell and Weinert, 1989). One of the best characterized pathways in *S. cerevisiae* is induced by DSBs and involves a cascade of rapid phosphorylations and possibly other post-translational modifications at the site of the damage. A first event is the phosphorylation of the histone H2A by Tel1, creating the substrate for the recruitment of additional checkpoint factors that allow the spreading of the platform over several kb along the chromosome (Downs et al., 2000; Shroff et al., 2004). In parallel with this, the RPA-coated ssDNA resulting from the 5′ to 3′ resection of the DSB serves as well for the loading of another set of checkpoint factors, including the complex formed by the ortholog of Tel1, Mec1, and Ddc2, the 9-1-1 checkpoint clamp composed of Ddc1, Rad17, and Mec3, assisted by the checkpoint clamp loader composed of Rad24 and Rfc2-5 proteins and Dpb11 (Lydall and Weinert, 1995; Zou and Elledge, 2003). Subsequently, Mec1 phosphorylates and activates the key checkpoint factor Rad9, which is separately loaded via an interaction with modified histones (Emili, 1998). Rad9 serves as a molecular adapter to amplify the checkpoint signal. It allows the phosphorylation of the main checkpoint effector Rad53, and/or in a minor way Chk1, by Mec1 (Sanchez et al., 1996; Schwartz et al., 2002). Phosphorylated Rad53 is then released and phosphorylates a set of substrates away from the site of damage that will interfere with the cell cycle progression to fulfill its arrest. Thus, in *S. cerevisiae*, although the DSB initiates all of the processes of checkpoint activation and chromatin modification at the site of the damage, the resulting ssDNA appears to be the major signal for Mec1 activation and cell cycle arrest. In mammalian cells, ATM appears to have a more prominent role in arresting the cell cycle.

During replication stress, Mrc1 (Claspin in humans), which travels with the replication fork, is thought to play the adaptor role in the Mec1-dependent activation of Rad53 (Osborn and Elledge, 2003). In this context, Rad53 activation requires both Rad9 and Mrc1 (Alcasabas et al., 2001). The subsequent activation of the replication checkpoint acts together with replisome stability factors to prevent fork collapse and probable resultant DSB, to facilitate fork restart (Tourriere and Pasero, 2007). Mrc1 has a pivotal role because it acts as a checkpoint and as a replication fork stability factor. The exact structure(s) that trigger the phosphorylation cascade is uncovered, although accumulation of ssDNA has been detected at replication forks when challenged by genotoxic agents providing a platform for the checkpoint to amplify (Sogo et al., 2002). Recently it was proposed that topological constraints could also contribute to activating the checkpoint (Bermejo et al., 2011). Nevertheless, the events accompanying the stabilization of fork stalls, their reactivation, or their repair appear to be multiple, and this area has been the subject of active investigations (Branzei and Foiani, 2010).

Once repair is complete, the checkpoints are turned off in a process called recovery (Harrison and Haber, 2006; Clémenson and Marsolier-Kergoat, 2009). First, checkpoint stimuli are expected to decrease as DNA is repaired. Second, a series of phosphatases, including Ptc2, Ptc3, and Pph3, revert the phosphorylated state of several key players and targets of the checkpoint (Leroy et al., 2003; Keogh et al., 2006). Also, when DNA damage is irreparable, a related process called adaptation occurs, in which cells resume cell cycle after a prolonged period of arrest (Sandell and Zakian, 1993).

To summarize, the DNA damage checkpoint pathway is composed of a cascade of phosphorylations composed of sensors, mediators, and effectors, first focused at the sites of DNA damage. The initial signal can be the DSB itself, ssDNA, or perhaps topological constraints or other structures. Finally, the factors involved either contribute in a unique way (they are essential) or act synergistically to obtain a robust cell cycle arrest in the presence of DNA damage (they are redundant or overlapping). In the next section, we will describe the steps of these pathways that were found to be involved in telomere biology.

## Are Short Telomeres Detected as DNA Damage in *S. cerevisiae*?

**Figure 4:**
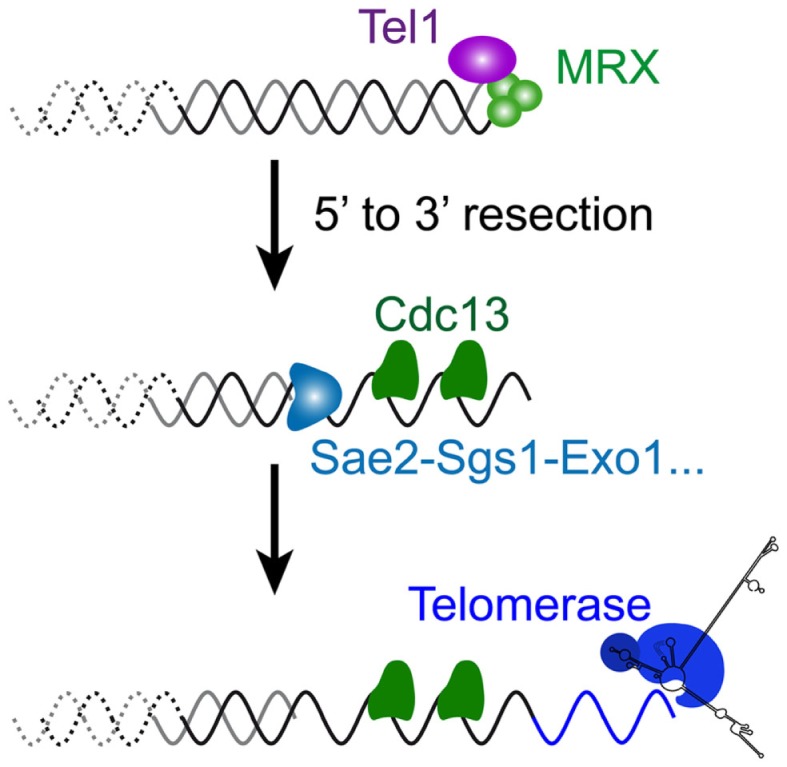
**Molecular response to a short telomere in the presence of telomerase in *S. cerevisiae***. The very first steps of DSB processing shown in Figure 3 also operate at short telomeres of wild-type cells, since a similar 5′–3′ resection takes place late S, by the time telomeres are replicated (Figure 2D). Coated with the telomere-specific Cdc13, the resultant overhang structure is required for the recruitment of telomerase to ensure telomere length homeostasis.

The relationship between telomeres and the DNA damage repair machinery is intricate and still far from being understood. Concomitant with the discovery of the main telomere function, which is to inhibit checkpoint activation (Sandell and Zakian, 1993), the involvement of DNA damage checkpoint proteins in telomere maintenance itself was discovered (Lustig and Petes, 1986; Greenwell et al., 1995; Ritchie et al., 1999). First it was found that *tel1*Δ cells display very short telomeres (about 100 bp instead of 300 bp), whereas *mec1*Δ effects are more modest. Mec1 is not essential in maintaining telomere length on its own but becomes essential in the absence of Tel1: a double mutant *tel1*Δ *mec1*Δ is unable to maintain telomeres and cells progressively lose viability, similar to telomerase-deficient cells (Ritchie et al., 1999; Chan et al., 2001). This indicates that these checkpoint kinases are required for telomerase elongation. Now it is known that the complex that recognizes DSBs comprising MRX and Tel1 associates with short telomeres by the time they are replicated (Ritchie and Petes, 2000; Tsukamoto et al., 2001; Hector et al., 2007; Sabourin et al., 2007; Sabourin and Zakian, 2008). Similar to a DSB, this binding results in a 5′–3′ resection that generates the long and transient 3′ overhangs at or after the passage of the replication fork, likewise depending on Sae2, Sgs1, Dna2, and Exo1 (Wellinger et al., 1996; Larrivee et al., 2004; Bonetti et al., 2009) (Figures 2D and 4). In contrast to DSBs, this telomeric ssDNA does not activate Mec1 (McGee et al., 2010), nor does it stimulate the downstream DNA-repair factors. This is probably because of Cdc13, which binds to single-stranded telomeric DNA, thus limiting the recruitment of RPA in this region. The limited recruitment of RPA is consequently presumed to be insufficient for the activation of Mec1 or the recruitment of Rad52 and the HR machinery. Instead, telomerase is recruited and elongates the short telomeres (Teixeira et al., 2004; Goudsouzian et al., 2006; Arneric and Lingner, 2007; Bianchi and Shore, 2007). Therefore, early steps of short telomere processing during replication share striking similarities with DSB processing, but the products are noticeably different (Figure 4). Although mammalian telomeres associate with MRN, ATM, and ATR (Verdun et al., 2005; Verdun and Karlseder, 2006), evidence for telomerase dependency on ATM and/or ATR activities is lacking (Feldser et al., 2006; McNees et al., 2010). However, many of these events are conserved in the distantly related yeast *S. pombe* (Moser et al., 2011; Yamazaki et al., 2012), indicating that a cell cycle-compatible, moderate activation of checkpoints could be a conserved feature of short telomeres to recruit telomerase. What happens to telomere processing if telomerase is not there?

A close analysis of telomerase-negative yeast cells rapidly indicated that the response to short telomeres was complex (Enomoto et al., 2002; Ijpma and Greider, 2003). When telomeres shorten in the absence of any component of the telomerase holoenzyme (TLC1, the RNA component of telomerase; Est2, the catalytic subunit; or the Est1 or Est3 subunits), cell culture growth rates decrease progressively in a few days of continuous liquid growth corresponding to about 60–80 population doublings (Lundblad and Szostak, 1989; Singer and Gottschling, 1994; Lendvay et al., 1996). When these cultures were streaked on plates at different time points during senescence, colonies became smaller and smaller and irregular in shape (Enomoto et al., 2002). Inspection of cells under the microscope showed a general increase in cell volume as cells reached the proliferation limit. Although they were not dividing, these cells were metabolically viable, as assessed with a vital staining (Ijpma and Greider, 2003). Furthermore, strains with longer initial telomere lengths can sustain additional population doublings in the absence of telomerase, further supporting the idea that telomere length determines proliferation potential (Smolikov and Krauskopf, 2003; Lebel et al., 2009).

Because telomeres shrink in these cultures, one obvious hypothesis to explain the decrease in growth capacity is that erosion reaches subtelomeres and ultimately internal genes, which would be incompatible with sustained cell divisions. To rule this out, chromosome instability was measured in telomerase-deficient cells (Ijpma and Greider, 2003). An artificial non-essential chromosome was introduced in these cells and its loss was evaluated with genetic markers placed along the chromosome. The authors found that the modest increase in the rate of chromosome loss did not correlate with the high loss of viability, suggesting that the loss of pieces of chromosomes was not the major cause of cell proliferation arrest.

Strikingly, in senescing budding yeast cultures, an accumulation of cells arrested in G2/M is observed (Enomoto et al., 2002; Ijpma and Greider, 2003). The analysis of spindle length indicates that cells are arrested before the metaphase-to-anaphase transition. Large cells, dubbed “monster cells,” also appear that contain several buds and condensed DNA (Enomoto et al., 2002). Overall, senescence appeared to be like a permanent DNA damage checkpoint activation. The phenotype of G2/M arrest was strictly dependent on Mec1, Rad24, Mec3, and Ddc2. This suggests that the absence of telomerase indeed leads to a checkpoint similar to DSBs (Enomoto et al., 2002; Ijpma and Greider, 2003) (see Table 2). Interestingly, these checkpoints factors are required for viability at early stages of senescence, because small colonies could be obtained by the plating of early senescing cultures lacking checkpoint factors (Enomoto et al., 2002). This indicates that telomerase deficiency may generate moderate genomic instability even at the early stages of senescence, before telomeres reach a critical length, and that DNA damage checkpoints might protect these cells. A possible explanation is that telomerase is required to elongate a small subset of critically short telomeres resulting from breaks during semi-conservative DNA replication (Miller et al., 2006; Chang et al., 2007; Sfeir et al., 2009). In contrast, deletion of *TEL1* in a *tlc1*Δ background does not suppress the progressive accumulation of G2/M cells to the same extent (Ritchie et al., 1999; Enomoto et al., 2002; Ijpma and Greider, 2003; Grandin et al., 2005; Abdallah et al., 2009). Rather, the decrease in colony size as cultures senesce, observed for *tlc1*Δ strains, is delayed. This indicates that, although it is not essential for a complete G2/M arrest, Tel1 promotes the signaling of eroded telomeres. Likewise, in the case of DNA damage, the ability of Tel1 to achieve cell cycle arrest is visible in *S. cerevisiae* only in the absence of Mec1 (Nakada et al., 2003; Grenon et al., 2006; Mantiero et al., 2007). Thus, Tel1 and Mec1, two structurally related kinases, display similar and overlapping biochemical activities but not equivalent biological roles in wild-type cells.

**Table 2:** **Role of the DNA damage checkpoint components tested so far in replicative senescence in *S. cerevisiae***.

Mutation	Gene orthologs in mammals	Phenotype in *S. cerevisiae* telomerase-negative cells			
		G2/M arrest	Progressive loss of viability	Rad53 activation	Reference
*tel1*Δ	ATM	+	Delayed	+	Ritchie et al. (1999), Enomoto et al. (2002), Grandin et al. (2005), Abdallah et al. (2009)
*mec1*Δ *or* *ddc2*Δ	ATR	−	Delayed	−	Enomoto et al. (2002), Ijpma and Greider (2003), Abdallah et al. (2009)
*rad24*Δ	RAD17	−	Delayed	−	Ijpma and Greider (2003)
*ddc1*Δ *or* *mec3*Δ	RAD9 or HUS1	−	Delayed	−	Enomoto et al. (2002), Grandin et al. (2005)
*rad9*Δ	53BP1; BRCA1; MDC1	+/−	Delayed	+/−	Enomoto et al. (2002), Ijpma and Greider (2003), Grandin et al. (2005)
*mrc1*Δ	Claspin	+	Accelerated	+/−	Grandin et al. (2005), Grandin and Charbonneau (2007)
*rad53*Δ	CHK2	+/−	Delayed	n.a.	Enomoto et al. (2002), Grandin et al. (2005)

*n.a., not applicable*.

The role of Rad9 in senescence was more laborious to unravel. Although the G2/M arrest was found to occur in telomerase-deficient cells lacking Rad9, indicating that it is dispensable for the short telomere checkpoint (Enomoto et al., 2002), this factor appeared to be play a role, according to independent works (Ijpma and Greider, 2003; Grandin et al., 2005). To address this discrepancy, several methods to quantify the relative importance of the different actors were employed. First, Rad53 was found to be phosphorylated during the course of senescence, as measured in electrophoretic mobility shift on western blots, and this phosphorylation was affected by the depletion of either Mec1, Rad9, or Rad24 (Ijpma and Greider, 2003; Grandin et al., 2005). Using the self-phosphorylation of Rad53 as a second readout for checkpoint activation, it was found that, in fact, Rad53 activation required both Rad9 and Mrc1 (Grandin et al., 2005). This indicates that in addition to a canonical DSB checkpoint, a replication stress could occur at eroded telomeres. Accordingly, both Rad9 and Mrc1 were found to be phosphorylated in the course of senescence (Grandin et al., 2005). Therefore, short telomeres appeared to be detected as DNA damage, both as a DSB and as a replicative stress.

The role of Mrc1 at telomeres is further complicated by the observation that *MRC1* deletion does not delay senescence or suppress the G2/M arrest, as expected for a checkpoint (Grandin et al., 2005; Grandin and Charbonneau, 2007). Instead, *mrc1*Δ *tlc1*Δ cells senesce earlier than *tlc1*Δ with the appearance of the G2/M arrest within fewer generations. Although *mrc1*Δ cells have slightly shorter telomeres (like other checkpoint mutants), telomere-shortening rates were comparable in both genetic contexts, indicating that the telomeres were less protected or triggered more signals in the double mutant. This is in accordance with genetic studies showing that Mrc1 has an additional checkpoint-independent role in protecting telomeres (Grandin and Charbonneau, 2007; Tsolou and Lydall, 2007). A plausible explanation that embraces all of these observations involves the dual function of Mrc1 in both replication checkpoint and replisome stability, namely in coordinating DNA polymerase and DNA unwinding during replication (Tourriere and Pasero, 2007). In accordance with this interpretation, a Rad53 allele defective in kinase activity also accelerates senescence of *tlc1*Δ cells (Lee et al., 2007).

Finally, the downstream targets of this checkpoint activation appear to be the same as for DNA damage: a gene encoding a component of the ribonucleotide reductase is activated via Dun1 phosphorylation and the G2/M arrest reveals the operative interference with the cell cycle progression (Ijpma and Greider, 2003; Grandin et al., 2005).

To conclude, at first glance, the checkpoint pathway activated in the absence of telomerase appears to be similar to a DSB. However, this response is modulated by the partial phenotypes found in *rad9*Δ background and evidence of the involvement of Mrc1. Thus, the telomere must signal both a DSB and a replication stress (Grandin et al., 2005). An interesting possibility for this dual signaling is that it might occur at different moments of telomere replication and/or at distinct telomeres. Some telomeres may experience a replication default activating Mrc1, and in the absence of telomerase to repair, the replication fork stalls, and eventually breaks and signals as a DSB. Other telomeres, because they lack sufficient telomeric proteins, simply signal as a DSB.

A subsequent question is whether telomere-less ends are sufficient to trigger the signal or whether other events occurring at other sites of the genome contribute to this signaling? Also, is a dysfunctional telomere (because of the lack of telomeric factors, for instance) inducing a similar signal?

## Short Telomeres Due to the Absence of Telomerase are Different from Dysfunctional Telomeres Due to Lack of Protection

Telomeres can be altered in numerous ways: alteration of telomeric sequences and mutations in telomeric proteins both lead to telomeres with a different proteome and perhaps a different transcriptome that sometimes is incompatible with cell life. For example, mutations in the telomerase RNA template region lead to a variety of different classes of phenotypes, including growth arrest for the most severe (Lin et al., 2004). Curiously, these growth arrests occur at mitosis, very similar to senescence. However, this arrest is not dependent on the DNA damage checkpoint.

Another example is the allele *cdc13-1* of *CDC13* encoding the major telomeric ssDNA binding protein (Weinert and Hartwell, 1993; Wellinger and Zakian, 2012). This allele confers growth defects for temperatures higher than 25°C, and at non-permissive temperatures, cells arrest in G2/M and display a robust Rad53 phosphorylation. This is due to an Exo1-dependent 5′–3′ resection of telomeres that exposes subtelomeric ssDNA and therefore activates Mec1 through RPA binding (Garvik et al., 1995; Booth et al., 2001; Maringele and Lydall, 2002). The checkpoint is *MEC1*- and *RAD9*-dependent, similar to what occurs in senescent cells. However, in contrast to the situation in telomerase-deficient cells, no trace of Rad53 activation was found in *rad9*Δ cells, suggesting that Mrc1 is dispensable for checkpoint activation in *cdc13-1* cells (Grandin et al., 2005). This indicates again that the defect found in a *cdc13-1* genetic background is different from the one found at eroded telomeres due to the absence of telomerase. The fact that the *cdc13-1* checkpoint is not dependent on Mrc1 further indicates that the telomere defect of *cdc13-1* telomeres does not involve the replisome. It is possible that the defect caused by *cdc13-1* is dominant, activating a ssDNA-dependent checkpoint before telomeres are replicated.

The Ku complex, which was first described as crucial for NHEJ, was found to interfere with telomere biology at several levels (Wellinger and Zakian, 2012). What is striking is that yeast cells lacking the Ku complex also display partially uncapped telomeres. Again, the cellular response appears to differ from that of eroded telomeres in the sense that Mad2, a component of the spindle checkpoint, is activated in Ku-deficient telomeres, whereas this factor is not involved in senescence (Ijpma and Greider, 2003; Dewar and Lydall, 2011).

Therefore, altering the telomeric structure triggers a cellular response that is generally different from telomere shortening in the absence of telomerase. Accordingly, partially uncapped or unprotected telomeres are more vulnerable to senescence, as illustrated by the accelerated senescence observed in many mutants of telomere protein components (Nugent et al., 1996, 1998; Chang et al., 2011b).

## Senescence Checkpoints Emanate from the Shortest Telomere(s) in Senescent Cells

An important issue raised by checkpoint activation during senescence is whether it stems from the telomeres themselves or from a consequence of genome instability triggered by telomere dysfunction when telomeres erode. For instance, the telomeric cap structure is important for preventing telomere fusions in many species (Marcand et al., 2008; Jain and Cooper, 2010), and in budding yeast telomeres are more prone to NHEJ in the absence of telomerase (Chan and Blackburn, 2003; Mieczkowski et al., 2003). Thus, as they erode, telomeres may become more prone to fusions, leading to dicentric chromosomes and random DNA breaks in the subsequent mitosis, causing cell unviability.

In order to understand the impact of short telomeres on genome stability, Carol Greider and colleagues devised a system to measure genome stability upon transient exposure to telomerase depletion and progressive telomere shortening (Hackett et al., 2001; Hackett and Greider, 2003). In a strain in which *EST1* was mutated, they engineered a yeast-dispensable chromosome to contain several genetic markers that could reveal the remaining chromosome structure after telomerase depletion. They found that chromosome rearrangements in telomere-proximal regions increased up to 10^−4^ after prolonged exposure to telomere shortening, compared to 10^−6^ for such events in isogenic telomerase-positive cells (Hackett et al., 2001). These rearrangements were often non-reciprocal translocations and could be explained by the healing of terminal deletions by break-induced replication (BIR), a DNA-repair pathway in which a unidirectional replication fork copies a donor template to the end, reconstituting a novel telomere (Llorente et al., 2008). These events, although rare, were increased 100-fold in cells with very short telomeres, suggesting that these latter cells initiate a process of genomic instability that may occur very early in tumorigenesis, in which telomeres are supposed to be critically short. It is important to note that genome instability was not equally distributed along the chromosome. Instead, a gradient was observed, with sequence losses being more frequent at the ends of the chromosomes rather than at internal sites. This strongly suggests that genomic instability occurs near telomeres and is not a secondary consequence of a general genomic instability induced by telomerase loss or short telomeres. Therefore, the checkpoints activated during senescence likely emanate from the telomeres themselves.

Because telomeres can adopt different states within the cells, at least with respect to Tel1 and telomerase recruitment (see above), it is reasonable to think that not all telomeres will initiate the checkpoint activation at the same time during senescence. So the next logical question is to know whether a single or a small subset of the shortest telomeres adopts more frequently a checkpoint-activatable state. In order to address this issue, a cellular setting was generated in which a single telomere was critically shortened in a strain lacking telomerase activity (Abdallah et al., 2009; Khadaroo et al., 2009). The striking observation is that the presence of a single critically short telomere accelerates the onset of senescence. This suggests that a single very short telomere is sufficient to initiate earlier on the process of checkpoint activation. Because this telomere could be tracked, immunoprecipitation of chromatin could be performed to determine whether checkpoint factors are enriched. It was found that Tel1 and Mec1 are enriched at the critically short telomere and that Tel1 enrichment is not dependent on Mec1. Finally, Mec1 is required for the accelerated loss of viability in the presence of the short telomere, suggesting that the short telomere is recognized by Mec1 to initiate the checkpoint activation. Therefore, like in human fibroblasts, checkpoints are activated at the shortest(s) telomere(s) in the cell undergoing senescence.

## What Causes the DNA Damage Checkpoint Activation at Eroded Telomeres?

The fact that Mec1 is activated in senescence suggests that ssDNA is accumulated at the most eroded telomeres. Single-stranded telomeric DNA already arises during normal telomere replication, as mentioned. Thus, it is possible that in telomerase-negative cells, Tel1 accumulation at short telomere(s) stimulates resection. In addition, short telomeres are less prone to inhibit 5′ resection (Negrini et al., 2007). The combination of both effects would then result in increased resection of one or few telomeres in the cell. This would subsequently expose subtelomeric ssDNA, recruit RPA, and activate Mec1, as proposed (Abdallah et al., 2009; Khadaroo et al., 2009). However, the impact of Exo1 deletion in senescence was only a slight delay (Maringele and Lydall, 2002, 2004a; Hackett and Greider, 2003; Bonetti et al., 2009). Moreover, although telomeric and subtelomeric ssDNA was found to increase modestly and possibly transiently in telomerase-negative cells (Hackett and Greider, 2003; Deshpande et al., 2011), direct evidence for ssDNA accumulation at native short telomeres is currently lacking in both telomerase-positive and -negative cells. Therefore, accumulation of ssDNA at subtelomeres due to exacerbated telomere processing may not be the only signal for senescence, at least not for all cells.

In addition to ssDNA, some other structures may activate the replication checkpoint, namely some replication intermediates. Nevertheless, it is not obvious whether replication stress increases as telomeres erode or whether fork collapse arises rarely but randomly within telomeric sequences. In the latter case, the lack of telomerase activity to repair would generate rapid losses of telomeric repeats, contributing to the onset of senescence. Bidimensional gels assessing replication intermediates of telomeres show that *S. cerevisiae* telomeric repeats impose a replication pause up to 100 bp before the telomeric repeats (Makovets et al., 2004), in accordance with observations in other species (Miller et al., 2006; Sfeir et al., 2009). Of note is that this pause seems decreased at shorter telomeres, indicating that the loss of telomeric repeats suppresses the replication stress. In contrast, a similar independent analysis revealed an increase in *X*-shaped structures in *tlc1*Δ cells (Lee et al., 2007). Thus, the detection of a particular replication stress in a small subset of telomeres is probably challenging when one is probing a whole population of telomeres. Evidence that forks may be specifically processed when they reach DNA ends exists for induced breaks (Doksani et al., 2009), providing support for the hypothesis that the replication fork through shortened telomeres may indeed be specifically processed. Thus, at this stage, it is unclear how the replication checkpoint is activated in cells lacking telomerase.

## What Type of DNA Repair Response at Eroded Telomeres?

One way to address the structures involved in the signaling at the shortened telomeres is to analyze the cellular response in terms of DNA repair. Worthy of note is that both NHEJ and HR influence the viability of cells undergoing senescence, suggesting that both pathways operate at telomeres lacking telomerase-mediated maintenance, but in different ways.

In fact, it is plausible that telomere–telomere fusions theoretically contribute to the loss of viability of senescent cells. Yet evidence that general genomic instability stems from random breakage of dicentric chromosomes is lacking (see above). It remains that short telomeres are expected to display less Rap1 binding sites and that Rap1, the major telomeric protein, inhibits NHEJ at telomeres (Pardo and Marcand, 2005; Marcand et al., 2008). Moreover, as mentioned, a modest increase in fusions involving telomeres was detected in telomerase-deficient cells (Chan and Blackburn, 2003; Mieczkowski et al., 2003). Subsequently, the telomere fusions themselves would directly trigger the loss of viability. However, the role of NHEJ at telomeres was first shown to be minor in *S. cerevisiae* cells lacking telomerase. Deletion of *DNL4/LIG4*, which encodes the ligase IV, the major ligase involved in NHEJ in *S. cerevisiae*, was found to have no effect on senescence rates or on the genomic instability found at telomere-proximal regions in senescent cells (Hackett et al., 2001). Then, telomeres with reduced binding sites for Rap1 were obtained by mutating the template region of telomerase RNA. Such strains displayed very long telomeres because telomerase is not inhibited at these telomeres. Strikingly, when telomerase was removed in these cells, senescence was not accelerated. This suggests that telomeric Rap1 is not essential for the growth of telomerase-deficient cells and consequently, that the loss of viability likely does not stem from telomere fusions (Smolikov and Krauskopf, 2003). In contrast to these results, in an independent work, deletion of *DNL4* was found to suppress growth defects of *est2*Δ cells, indicating that growth defects of senescing cells are due in part to fusion events between telomeres (Meyer and Bailis, 2008). The discrepancy in these data obtained in different strain backgrounds could be due to a difference in the ability of NHEJ-dependent dicentric telomeres to be resolved. Indeed, dicentrics are resolved preferentially by breakage within the telomere fusions themselves (Pobiega and Marcand, 2010). This could also explain how telomere–telomere fusions increase genomic instability near telomeres and not randomly throughout the genome (Hackett and Greider, 2003). To conclude, NHEJ might fuse short telomeres, possibly in the G1 phase of the cell cycle. Then, depending on the genetic background, telomere–telomere fusions could break in the next mitosis, regenerating the original telomeres. However, this should also contribute to cell inviability when breakage occurs at non-telomeric regions of the dicentric chromosome, generating a DSB. Whether the resolution of these dicentrics contributes to the G2/M delay and to the activation of checkpoints, characteristics of senescence, should be carefully tested in future experiments.

The involvement of HR in senescence is also the focus of active investigations, and one has to distinguish between an early response of HR factors to eroded telomeres from a later response operating in the post-senescent survivors.

Post-senescent survivors were first observed after a prolonged time in culture, when a small subset of senescent cells could reenter a proliferative state (Lundblad and Blackburn, 1993). They maintained telomeres mainly through an HR-based mechanism (Le et al., 1999; Teng and Zakian, 1999), but other less canonical mechanisms were found to operate to protect and maintain chromosome ends with more or less efficiency (Maringele and Lydall, 2004b; Grandin and Charbonneau, 2009; Lebel et al., 2009; Wellinger and Zakian, 2012). The survivors are thought to arise at a rate of up to 10^−4^, but this is probably very variable, and actual precise values are lacking. For instance, mating type influences the frequency of emergence of survivors: cells in which silencing at the mating type loci is disturbed or haploid cells in which both *Mat*a and *Mat*α loci are coexpressed bypass senescence (Lowell et al., 2003; Meyer and Bailis, 2008). Also, the original telomere length greatly influences these rates, as longer telomeres favor the emergence of survivors, and, in *Kluyveromyces lactis*, a related budding yeast, the presence of a single long telomere allows the senescence to be bypassed (Topcu et al., 2005; Lebel et al., 2009; Chang et al., 2011b). So longer telomeres not only increase the global proliferative capacity of cells deficient in telomerase activity (Smolikov and Krauskopf, 2003; Lebel et al., 2009) but also potentially improve the fitness of cells in which an accidental repair such as BIR has occurred between a long and a critically short telomere. It is important to note that a survivor character is a recessive trait, as telomerase reintroduction in such strains immediately restores the telomerase-mediated mode of telomere maintenance (Makovets et al., 2008; Becerra et al., 2011). This indicates that telomerase represses recombination-based telomere maintenance, and, conversely, in its absence, recombination mechanisms might be upregulated. This raises the question whether, before the emergence of post-senescence survivors, the HR factors operate at eroded telomeres in the absence of telomerase and if so, what’s their role in protecting them from, or in promoting genomic instability. As we will describe in the next paragraphs, HR acts at telomeres after the removal of telomerase activity, yet its role is clearly not fully understood.

Rad52 is a key protein in the HR pathway. The best studied role of Rad52 is its activity in assisting the loading of Rad51 on RPA-coated ssDNA to initiate homology search, but Rad52 is also involved in many Rad51-independent pathways of DNA repair. Rad52 is also required for most pathways of telomerase-independent survival, but its role in earlier stages of senescence is still unclear. Indeed, *rad52*Δ cells deficient in telomerase activity display a strong synthetic growth defect from the moment telomerase activity is removed and well before post-senescent survivors emerge (Kass-Eisler and Greider, 2000; Hackett et al., 2001). The same was observed for *rad51*Δ and other gene deletions of the *RAD52* epistasis group. When fluorescent microscopy was used to visualize single cells, Rad52 was shown to form foci very early after telomere loss that co-localize with telomeres (Khadaroo et al., 2009). Thus, viability in the absence of telomerase greatly depends on HR, likely operating at the telomere level. If BIR, a branch of HR, was shown to occur at the telomeres (Hackett et al., 2001), its low frequency clearly does not explain all of the HR events at telomeres. Accordingly, most very short telomeres do not acquire novel telomere sequences from other telomeres, as assessed by the sequencing of individual telomeres (Abdallah et al., 2009). Therefore, it is possible that HR is acting mainly through exchanges between sister chromatids. In support of this, the deletion of *MMS1*, which encodes a subunit of an E3 ubiquitin ligase involved in replication repair and not in gene conversion at a DSB, has a similar phenotype than the deletion of *RAD52* (Abdallah et al., 2009).

It is interesting that the abnormal replication intermediates observed in *tlc1*Δ cells increased in *tlc1*Δ *sgs1*Δ double mutants (Lee et al., 2007). Analyses of these structures suggest that they are partially dependent on Rad52 and on the kinase activity of Rad53. In fact, Sgs1 not only has a function in the resection of DSBs and telomeres but also has a critical function in processing recombination intermediates that occur at replication fork stalls, as detailed recently (Vanoli et al., 2010). In accordance with this, the deletion of Sgs1 has a deleterious effect on the growth of telomerase-negative cells (Cohen and Sinclair, 2001; Johnson et al., 2001; Azam et al., 2006).

More recently, the Smc5/6 complex, which contributes to the removal of abnormal recombination intermediates that arise between sister chromatids during DNA replication and repair, was shown to sustain the growth of senescing cells (Chavez et al., 2010; Noel and Wellinger, 2010). This phenotype was mimicked by disruption of the SUMO-ligase activity of Mms21, which is associated with the Smc5/6 heterodimer. Moreover, the defect in growth is increased when combined with *RAD52* deletion, indicating partial independence in the mechanisms required to maintain telomeres. Analyses of individual telomere sequences further suggest that the complex is required to prevent catastrophic telomere sequence losses in a minor proportion of telomeres. These results, again, underline the importance of sister telomere interactions during replication and DNA-end processing, in both the presence and absence of telomerase.

**Figure 5:**
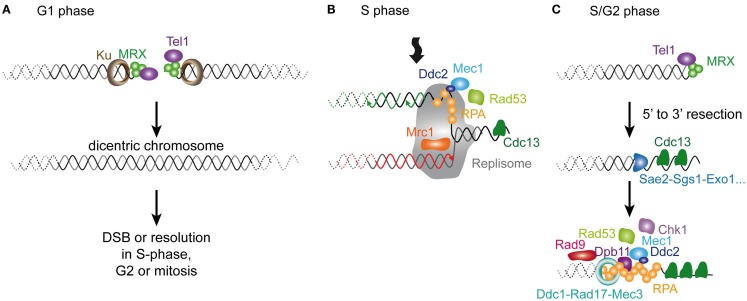
**Molecular response to a short telomere in the absence of telomerase in *S. cerevisiae***. In the absence of telomerase, three distinct, but non-exclusive scenarios can be envisaged. **(A)** In the G1 phase of the cell cycle, short telomeres incur a risk of being recognized by the DSB sensing factors. Although in budding yeast, this may not be sufficient to trigger a G1 checkpoint (Zierhut and Diffley, 2008; Symington and Gautier, 2011), this probably contributes to cell cycle arrest in G1 of senescing cells in mammals. The main risk in budding yeast cells is the NHEJ pathway and the formation of telomere–telomere fusions. This will pose a problem in the next S-phase, since palindromic telomeric fusions may generate a replication stress and subsequent activation of a replication checkpoint. Then, if fusions resist to replication, in the next mitosis, the segregation of dicentric chromosomes may contribute to a delay of the M phase. Their resolution may generate immediate unviability or, if it occurs within telomeric repeats, it recreates short telomeres with uncertain end structures. **(B)** Telomeres form natural replication pauses and in case of telomere breaks during replication, the preferential mode of repair is telomerase-dependent re-elongation. In the absence of telomerase, it’s not known whether replication is made more difficult at the shortest telomeres by a specific processing of DNA ends. Nevertheless, the activation of the replication checkpoint in senescent cells may reflect a myriad of different structures occurring at telomeres when replicated in the absence of telomerase. **(C)** Finally, short telomeres may be processed as DSBs, whereby an extended 5′–3′ resection may expose subtelomeric ssDNA resulting in Mec1 activation as in Figure 4. Note that all of the three scenarios can contribute to the G2/M delay or arrest observed in senescent cells.

To summarize, the DNA-repair pathways operating at telomeres after removal of telomerase include NHEJ, replication stress response and end resection, probably at different phases of the cell cycle (Figure 5). Concerning the replication stress, a question that remains unanswered is what portion of telomeres is subjected to such replication defaults. Again, whether these occur preferentially at short telomeres, as proposed (Lee et al., 2007; Abdallah et al., 2009), or whether they are a random event within telomeric sequences remains to be clarified. Notwithstanding, the implication of these pathways in yeast senescence complements investigations of the role of mammalian ReqQ helicases, recombination and replication factors at mammalian telomeres (Badie et al., 2010; Hagelstrom et al., 2010; Leman et al., 2012).

## Future Directions

To conclude, several pathways appear to be involved in the activation of the checkpoint that leads to a G2/M arrest in *S. cerevisiae* cells lacking telomerase. Although stemming from telomeres, several structures could converge to a similar cellular phenotype. Telomeres in a cell are all different in length, and perhaps in state, and may signal differently. Investigating the length dependency and the proteome associated with each type of signaling telomere would be a good start to address the variety of telomere states within a cell.

One striking aspect of replicative senescence is its permanent character. Checkpoints are maintained activated, as assessed by Rad53 phosphorylation, as mentioned above. Several non-exclusive phenomena may lead to this: (i) the structure(s) that activate(s) the checkpoint persist(s), (ii) repair is inhibited or ineffective, and (iii) recovery and/or adaptation is prevented. Concerning the first point, it was proposed that the structures activating the checkpoint may alternate keeping the checkpoint switched on (Deshpande et al., 2011). Alternatively, as they shorten, telomeres may switch to signaling states more frequently, as proposed previously (Blackburn, 2000). The permanent checkpoint status would then stem from a serial accumulation of checkpoint-activating structures. More data on the time in which individual yeast cells are arrested in G2/M are needed to estimate how the process of senescence is progressively established in a population. For the second point, we saw that HR acts at the telomeres, mainly through sister chromatid recombination, restricting the ability of telomeres to display a net elongation and subsequently limiting the efficiency of this pathway in terms of a gain in the proliferation capacity. Whether the use of other telomeres as a template is prevented by telomere-dependent structures, such as the telomeric heterochromatin, is unclear. Yet this question is obviously highly relevant to understanding the maintenance of genome stability in senescent human cells. Concerning the third point, adaptation and recovery from DNA damage checkpoints are underinvestigated in senescence. Although depleting an essential phosphatase in this process does not alter the global rate of senescence (Abdallah et al., 2009), it is possible that the proliferative capacity of individual cells is affected by these pathways. Addressing all of these points in future work should help improve understanding of the permanent character of senescence at the molecular level.

Another important point that will certainly be thoroughly investigated is the chromatin environment of telomeres in senescence. This is illustrated by the finding that a histone acetyltransferase and other telomeric chromatin components modulate senescence (Kozak et al., 2009). Telomeric transcription also influences senescence (Maicher et al., 2012; Pfeiffer and Lingner, 2012). One pathway involves the modulation of the shortening rate of telomeres in the absence of telomerase via an interference with chromosome end processing activities, namely the 5′–3′ resection. Future work will tell whether other pathways are also involved. In addition, it will be extremely exciting to integrate the processing of eroding telomeres within the nuclear volume. Indeed, telomeres travel back and forth across the bud neck in telomerase-deficient cells that are arrested in G2/M (Straatman and Louis, 2007), likely in connection with near nuclear pore complexes (Khadaroo et al., 2009). Understanding of the biological significance of this localization is in its infancy (Lisby et al., 2010).

Implications of telomere-mediated senescence on cellular physiology greatly benefit from genome-wide screens more easily implemented in *S. cerevisiae*. Recently, a synthetic growth genome-wide screen was performed to select for genes that affect growth when combined with telomerase deletion (Chang et al., 2011a). Further investigation of the tens of newly identified genes involved in telomere biology as well as in senescence will certainly open up novel avenues of research. Also, the transcriptome of a culture undergoing senescence and survival was analyzed (Nautiyal et al., 2002). Induction or repression of many genes was found that largely exceeded the ones expected from a simple DNA damage response. Notably, subtelomeric genes were found to be induced in accordance with a loss of telomeric silencing with shorter telomeres. However, the unexpected finding was the alteration of expression of genes involved in energy production, suggesting that despite glucose in the medium, yeast cells shift to aerobic respiration. Accordingly, mitochondria were found to proliferate when cells reached the lower growth rate. Determining whether these mitochondria are functional requires further studies, in particular from the perspective of the recent evidence of a direct link between mitochondria and telomeres in senescence in mice deficient for telomerase (Sahin et al., 2011).

## Conclusion

The search for a unique and common cause of aging has been challenging because multiple causes and likely many independent pathways influence the lifespan at the level of the organism. Accordingly, the aging process probably reflects a decrease in the turnover of molecules, in their repair or renewal. This can have many targets, as cells work as a whole, requiring all parts of the machinery to work properly. In this perspective, telomeres take on a special place because they consist of regions in the cell that may have evolved in certain species to control their own maintenance and repress their own repair under certain circumstances. Thus, the crosstalk between telomeres and the cellular cycling machinery is a privileged way for the cell to control its destiny as a function of its history. Yeast cells lacking telomerase, which produce telomere shortening as a cause of replicative senescence, share striking similarities with mammalian cells in their early cellular response, in particular in the early activation of a DNA damage checkpoint. Dissection of the telomeric processing that activates this checkpoint is obviously facilitated in yeast because of its easy manipulation. In addition, the vast knowledge on DNA-repair pathways in *S. cerevisiae* can be integrated into the telomere biology. Importantly, in a yeast cell culture population, most yeast cells are “young cells” with respect to accumulation to aging. Therefore, the cellular settings used in yeast mostly allow for the specific dissection of telomere-induced senescence, among the other vast consequences of aging. Hence, data obtained in budding yeast complement investigations using mammalian models to make rapid progress in this field so relevant for human health.

## Conflict of Interest Statement

The authors declare that the research was conducted in the absence of any commercial or financial relationships that could be construed as a potential conflict of interest.
